# Acne Vulgaris

**Published:** 1908-04-25

**Authors:** 


### Dermatology.

### ACNE VULGARIS.

Acne Vulgaris is a skin affection with very well marked features: it can seldom be confused with any other eruption. It is a disease of youth, occurring most commonly between the ages of fifteen and twenty-five; it attacks chiefly the face and the upper part of the back and the shoulders; the skin of these parts is always more or less greasy; the primary and essential lesion is the "blackhead," or comedone, plugging the mouth of the pilo-sebaceous follicle; there are also papules and papulo-pustules, the result of inflammation around the comedones.

It was formerly supposed that the comedones were the result of the plugging of the follicles by accumulated sebum and immature hairs. Modern research has shown that the comedone is made up of concentric layers of horny-cells enclosing a mass of micro-organisms (the *micro-bacillus of acne*). It seems probable that the oily seborrhœa is a result of hyperactivity of the sebaceous glands at puberty, and that the acne bacillus, finding in the fat at the mouth of the follicle not only a suitable soil but a sheltered place, here multiplies and grows into a colony. As a protective reaction the colony becomes encysted by the horny cells. The horny plug thus formed in the mouth of the follicle itself acts as a local irritant, and this gives an opportunity for the entry of the common staphylococcus of the skin, which results in the secondary inflammation in and around the gland and follicle, clinically apparent as the papule and the papulo-pustule.

In an ordinary case of acne, then, the essential features are the greasy skin, the "blackheads" or comedones, and a certain number of secondary papules and papulo-pustules—these lesions being situated on the face, neck, and shoulders of a young person of either sex. Patients may be anæmic and constipated, and they frequently suffer from cold hands and feet, but the most important factors in the ætiology of acne seem to be the period of puberty and a local microbic infection. In more severe cases the papulo-pustules may enlarge into deep-seated livid red indurated nodules, often very painful (acne indurata). They may contain a quantity of pus. If left alone, they may absorb, but generally they burst and leave ugly, irregular, sometimes pigmented, scars. Papules and papulo-pustules may occur on the face in bromide and in iodide eruptions, in syphilis, and in rosacea. Acne vulgaris is distinguished by the presence of comedones.

Most cases of acne will yield to treatment by local measures, if these are thoroughly and persistently carried out. These measures are: squeezing out the comedones by means of an expressor; nightly bathing with warm water and thorough soaping; followed by the application of a sulphur lotion. Since the comedone contains the micro-organism which is held to be the primary cause of the eruption, the expression of these plugs is most important, and the affected parts should be gone over carefully every day until all the "blackheads" are removed. No attempt, however, must be made to express the inflamed comedones. The soaping is best done with a shaving-brush. A suitable lotion for use after bathing and soaping is as follows:—

| | | |
|---|---|---|
| ℞ | Sulphuris præcip. ... ... ... ... | ʒij. |
| | Glycerini ... ... ... ... ... | ʒij. |
| | Spirit vini rect. ... ... ... ... | ℥ss. |
| | Etheris ... ... ... ... ... | ʒij. |
| | Aq. Rosæ ad ... ... ... ... | ℥viij. |

The lotion is to be mopped on freely and the powder allowed to dry on, and to remain until washed off the next morning. If the face gets sore the soap and lotion may be omitted for one or two evenings. Papulo-pustules when they appear should be incised with a very fine scalpel and afterwards bathed with hot water. Indurated nodules, in addition to being incised, should have a tiny drop of pure carbolic introduced into the cavity on a pointed match-stick. If anæmia or constipation be present these conditions must be treated. When suppuration is marked, calcium sulphide (in pills, gr. ij., thrice a day) may be given, or ceredin (yeast-fat) pills.

In severe or obstinate cases other treatment may be necessary. There are three methods—(1) a shelling paste; (2) staphylococcic vaccine; (3) *x*-rays.

(1) *Shelling pastes* are used largely on the Continent. They give excellent results, but require care in their application. Their use necessitates the patients' confinement to the house for a week or so.

1. *Unna's Schälpasta.*

| | | |
|---|---|---|
| ℞ | Zinci oxidi ... ... ... ... ... | ʒjss. |
| | Terræ siliceæ ... ... ... ... | gr. xv. |
| | Ol. benzoinali ... ... ... ... | ♏xl. |
| | Adipis benzoin. ad .. ... ... | ʒss. |
| | +Resorcin *i.e.* 10 per cent. to 50 per cent.) | |

This paste is applied twice daily for three or four days, beginning with 10 per cent. resorcin and increasing to 50 per cent. on the third or fourth day. During the rest of the week the superficial layers of the epidermis shell off like a mask, leaving the condition of the skin much improved. The process may be repeated after a time.

2. *Schwimmer's Pasta Decorticans.*

| | | |
|---|---|---|
| ℞ | Sulphuris præcip. ... ... ... ... | ʒj. |
| | β naphthol ... ... ... ... ... | ʒj. |
| | Saponis viridis ... .. ... ... | ʒij. |
| | Adipis præparat ... ... ... ... | ʒiv.-ʒvj. |

This paste is applied at night for several days in succession; it is allowed to remain in contact with the skin for from one to two hours, then washed off and a dusting-powder applied. In one or two weeks desquamation follows, and a soothing ointment is then prescribed.

(2) *Staphylococcic Vaccine.*—Wright's method may be employed in obstinate cases of pustulating acne. It is often successful, but not always.

(3) *X-ray Treatment* gives brilliant results, but should be reserved for obstinate cases. It is successful also in mild cases, but other methods will cure these. It must be applied with great care and in measured doses. Nodules, papules, comedones, and greasiness of the skin disappear. But sometimes there is a severe temporary exacerbation of pustulation, which cannot be foreseen.

